# Social capital for carers of patients with advanced organ failure: a qualitative exploration of stakeholders’ perspectives

**DOI:** 10.1186/s12889-024-18213-6

**Published:** 2024-03-02

**Authors:** Marques Shek Nam Ng, Winnie Kwok Wei So, Kai Chow Choi, Oluwadamilare Akingbade, Wallace Chi Ho Chan, Helen Yue Lai Chan, Carmen Wing Han Chan

**Affiliations:** 1 The Nethersole School of Nursing, The Chinese University of Hong Kong, Hong Kong, China; 2 Department of Social Work, Education and Community Wellbeing, Northumbria University, Newcastle upon Tyne, UK

**Keywords:** Social capital, Carer, Advanced organ failure, Stakeholder, Qualitative, Community, Healthcare

## Abstract

**Background:**

Carers of patients with advanced organ failure (AOF) experience a tremendous caregiving burden. Social capital utilizes the internal strength of a community to support its members and may provide carers with comprehensive support. This study aimed to identify the different sources of social capital that can support carers of patients with AOF from the perspectives of stakeholders*.*

**Method:**

A descriptive qualitative study was conducted in community settings from April 2021 to May 2022. Stakeholders from medical social work departments, self-help groups, and non-governmental organizations were recruited, while some community members were invited through online media platforms. Individual semi-structured interviews were conducted using an interview guide. Interview transcripts were analyzed using a qualitative description approach. In total, 98 stakeholders, including 25 carers, 25 patients, 24 professionals, and 24 community members, were recruited using purposive and snowball sampling.

**Results:**

Six categories about social capital for carers emerged, namely, carer attributes, the community, social care services, healthcare services, information, and policies. While the attributes of carers and their relationships with care recipients had a significant influence on caregiving, support from different groups in the community, such as neighbors and employers, was valued. Good communication of information about caregiving and social services was emphasized as being helpful by carers and other stakeholders. While carers presented a need for various healthcare and social care services, several features of these services, including their person-centeredness and proactive reach, were deemed useful. At the societal level, policies and research on comprehensive supportive services are warranted. The different sources of social capital constitute a multi-layer support system in the community.

**Conclusion:**

Carers can utilize personal attributes, interpersonal relationships, community resources, and societal contexts to enhance their caregiving. While this system can serve as a framework for building carer-friendly communities, interventions may be required to strengthen some aspects of social capital.

**Supplementary Information:**

The online version contains supplementary material available at 10.1186/s12889-024-18213-6.

## Introduction

The global impact of non-communicable diseases is expanding, and in 2019, they affected more than 7 billion people and caused 42 million deaths worldwide [1]. Individuals diagnosed with advanced organ failure (AOF), including chronic obstructive pulmonary disease (COPD), kidney failure (KF), and heart failure (HF), experience serious symptoms, a progressive decline in function, and psychosocial issues [2]. With a relatively long disease trajectory, these patients and their carers have significant burdens yet to be addressed. The term carers refers to “individuals who provide care to those who need it in the context of a relationship [3].” These carers, who are unpaid and often must engage in employment, are responsible for the daily care and health management (e.g., lifestyle modifications, medical treatments) of the patients [4]. The care of patients with AOF is more challenging than the care of those with other non-communicable diseases due to the complexity of the disease. Therefore, the AOF caregiving burden is equivalent to the caregiving burden of looking after patients with cancer [5] and is associated with various physical and mental health problems [6, 7]. Because of their prolonged engagement in caregiving, carers are often socially isolated [8, 9] and lack sufficient social support [10, 11].

Social capital refers to the standards, relationships, and norms that promote interactions within a community. It is the internal strength of a community that facilitates its members fulfilling their social role [12]. According to the World Bank [13], the different characteristics of the community, both structural (tangible) and cognitive (intangible), connect individuals and social groups within and across social levels for better mobilization of resources. These connections can be differentiated as bonding, bridging, and linking capital (Table 1). Evidence suggests that social capital may promote the physical and mental health of community-dwelling older individuals [14, 15]. In the case of caregiving, many carers spend most of their time at home with the care recipients, gradually reducing their investment in social capital [16]. In one study, [17] a lower level of social capital was associated with a worse caregiving experience. Therefore, it is reasonable to hypothesize that social capital, which mobilizes the internal resources of a community, leads to more comprehensive support for carers [10]. By sharing the responsibility of carer support among community members, the sustainability of the social care system can be improved.

**Table 1:** Different Forms of Social Capital

*Forms*	*Descriptions*	*Examples of Structural Capital*	*Examples of Cognitive Capital*
Bonding	Ties connect people who share similar demographic characteristics	Carer group	Relationship within family
Bridging	Horizontal ties that bridge people with broadly comparable socioeconomic status	Community support services	Relationship between carer and professional
Linking	Vertical ties between people with lower socioeconomic status and people in positions of influence in formal organizations	Government policy on carer support	Value in community about caregiving

In Hong Kong [18], the development of social capital commenced with the establishment of pressure groups and community-based consultative agencies, such as the District Councils, in the 1970s. These social structures have played a crucial role in engaging community members in social matters. However, most supportive services for carers have been provided by the government in collaboration with various non-governmental organizations (NGOs; Table 2) [19]. To address the evolving needs of vulnerable groups, the Community Involvement and Inclusion Fund was introduced in 2002 to foster social capital within communities. As a result, there are ongoing discussions on how to mobilize specific forms and sources of social capital to provide support for individuals in need. However, despite the significance of social capital to these individuals, as well as society, research has mainly focused on the perceived social support for carers [9, 20, 21].

**Table 2:** Caregiving Policy in Hong Kong

*About Hong Kong*	
• A special administrative region of China • A high-income economy (Gross domestic product in 2021: 369.2 billion USD)	
*About Caregiving Policy*	
• The local government largely subsidizes healthcare and social care services. • By partnering with non-governmental organizations, the government provides some supportive services to carers (e.g., respite care, caregiving training, and financial assistance) through elderly and rehabilitation service units.	

Information about availability of social capital that facilitates caregiving remains limited. This study aimed to identify sources of social capital that can be used to support the carers of patients with AOF from the perspectives of stakeholders.

## Materials and methods

This was a descriptive qualitative study conducted as part of a concept-mapping project [22]. In the initial steps, to invoke ideas for concept map creation, stakeholders were invited to share qualitative descriptions of their views on social capital. Given the diverse backgrounds of these stakeholders, individual semi-structured interviews were used to capture their perceptions of social capital for carers.

### Settings and participants

This study was conducted in community settings across Hong Kong from April 2021 to May 2022. Four categories of stakeholders, namely, carers, patients, professionals, and community members, were recruited using purposive and snowball sampling. The sample size was estimated based on the principles of data saturation [23] and concept mapping research [24]. Participants were recruited through collaborating organizations, including medical social work departments, self-help groups, and NGOs. To recruit community members, the team advertised the study through social media (e.g., Facebook). Experts who possessed knowledge of and/or experience in caregiving were identified via websites and publications and were invited to participate as community members. All participants provided their written consent to participate.

### Data collection

After each participant consented to participate, the research assistant (RA) made an appointment with the participant for an individual semi-structured interview. Because of concerns about the COVID-19 pandemic, participants could opt for virtual (i.e., web conferencing) or face-to-face interviews. All interviews were conducted in a quiet and private environment (e.g., home or interview room). The interviews were conducted using an interview guide (see Supplementary Material 1). For each interview, a brief description of social capital was provided to facilitate brainstorming. The interviewer then began the interview with a general request, “Please generate some short sentences or phrases that describe the resources in the community, including but not limited to medical and social services, that facilitate caregiving of patients.” Follow-up questions regarding different sources of support were used to elicit views. All interviews were audio-recorded. The interviews were conducted by the principal investigator (PI), a nurse researcher specializing in mixed-method research, and/or the RA, a psychology graduate who had received relevant training. Interview recordings were regularly reviewed by the research team to determine data saturation and ensure consistency.

### Data analysis

Descriptive statistics (i.e., mean, standard deviation, frequency, and percentage) were used to analyze the participants’ demographic backgrounds using the SPSS 25.0 software package (IBM Corp., Armonk, NY, USA). Interview recordings were transcribed verbatim by the RA and validated by the PI. The transcripts were analyzed using a qualitative description approach [25]. For the first 20 transcripts, two coders (the PI and RA) independently read the transcripts and highlighted content relevant to social capital. A codebook grouping similar content was developed through discussion and mutual agreement. The PI and RA then coded the subsequent transcripts using the code book. Any new codes that evolved in subsequent transcripts were discussed among the coders before they were added to the code book. The coders examined the codes to determine if they corresponded to any form of social capital (i.e., bonding, bridging, and linking) as defined by the World Bank [13]. After coding all the transcripts, the PI further grouped the codes into categories according to their sources. These categories were reviewed by the research team for their trustworthiness. Coding was conducted using the NVivo 11 software package (QSR International, Melbourne, Australia).

### Trustworthiness

Based on the framework of Lincoln and Guba [26], measures were taken to ensure trustworthiness. Two coders (the PI and RA) conducted data analysis, and their interpretations were discussed and validated by the research team. A summary of the findings was sent to participants for comment. These procedures involved investigator triangulation to achieve credibility. In terms of transferability, the backgrounds of the study and participants are described in this report. Contextual factors that may affect interpretation are discussed as limitations. During the data collection and analysis, notes were taken and filed to ensure dependability and confirmability.

### Ethical considerations

This study was approved by the Survey and Behavioural Research Ethics Committee of The Chinese University of Hong Kong (reference number: SBRE-20-714) prior to participant recruitment. Written consent was obtained from every participant before data collection, and the right to withdraw from the study at any point was guaranteed. Confidentiality was protected for all participants via the non-disclosure of identity. Codes were used to refer to participants in research records and reports. All research materials, including audio recordings and written information, were locked in a secured cabinet or electronic device. They will be discarded appropriately 5 years after the completion of the study.

## Results

Ninety-eight participants, including 25 carers, 25 patients, 24 professionals, and 24 community members, were recruited. Their demographic background is presented in Table 3. The majority of the carers (*n* = 25) included in the study were female (60%), aged greater than 40 years (72%), married (56%), and employed (52%). Almost half of them had received a tertiary education (48%), and 40% earned a monthly household income of less than HKD10,000 (approximately USD1,270). Of note, 60% of them were children of the patients. Almost half (48%) of the carers were caring for a patient with KF, and the mean duration of their engagement in caregiving was 9.6 years. The majority of patients (*n* = 25) included in the study were female (60%), and the most common diagnosis was COPD (48%), followed by KF (44%). The mean time since diagnosis was 14.2 years. Nurses (50%) and social workers (38%) constituted most of the carers with professions, and they had experience of caring for these patients for an average of 15.6 years. Many of the community members were staff members of local tertiary institutions (33%) or NGOs (25%) who provided care for other carers (e.g., carers of older individuals).

**Table 3:** Demographic Background of the Participants (*n* = 98)

*Characteristics*	*Total (n = 98)*	*Carers (n = 25)*	*Patients (n = 25)*	*Professionals (n = 24)*	*Community members (n = 24)*
Males	33 (34%)	11 (44%)	10 (40%)	8 (33%)	4 (17%)
Age (years)					
18–29	19 (19%)	4 (16%)	0 (0%)	6 (25%)	9 (38%)
30–39	17 (17%)	3 (12%)	0 (0%)	5 (21%)	9 (38%)
40–49	14 (14%)	5 (20%)	1 (4%)	5 (21%)	3 (13%)
50–59	17 (17%)	5 (20%)	5 (20%)	7 (29%)	0 (0%)
60–69	18 (18%)	6 (24%)	9 (36%)	1 (4%)	2 (8%)
≥70	13 (13%)	2 (8%)	10 (40%)	0 (0%)	1 (4%)
Educational level					
Primary or below	9 (9%)	0 (0%)	9 (36%)	0 (0%)	0 (0%)
Secondary	31 (32%)	13 (52%)	14 (56%)	2 (8%)	2 (8%)
Tertiary	31 (32%)	12 (48%)	1 (4%)	9 (38%)	9 (38%)
Postgraduate	27 (28%)	0 (0%)	1 (4%)	13 (54%)	13 (54%)
Employed	–	13 (52%)	2 (8%)	–	–
Monthly income (HKD)^a^					
< 10,000	24	10 (40%)	14 (56%)	–	–
10,000–29,000	11	6 (24%)	5 (20%)	–	–
30,000–49,000	6	3 (12%)	3 (12%)	–	–
≥50,000	5	3 (12%)	2 (8%)	–	–
Diagnosis of care recipient					
COPD	–	5 (20%)	12 (48%)	–	–
KF	–	12 (48%)	11 (44%)	–	–
HF	–	8 (32%)	2 (8%)	–	–

^a^HKD10,000 is approximately equivalent to USD1,270

*COPD* chronic obstructive pulmonary disease: *HF* heart failure: *HKD* Hong Kong Dollar: *KF* Kidney failure

Ninety-eight semi-structured interviews were conducted with a mean duration of 23 minutes. Thirty-one codes emerged, and these codes formed six categories, namely, carer attributes, social care services, healthcare services, the community, information, and policies. Table 4 presents the codes and exemplary quotes.

**Table 4:** Codes and Exemplary Quotes

*Codes*	*Exemplary Quotes*
**Category 1: Carer Attributes**	
1. Sense of responsibility^Bo^	“I just take care of my parents as they took care of me when I was young. It’s something I need to do. It’s my responsibility.” (Carer 7)
2. Readiness for accepting support^Bo^	“They (carers) need to accept their situations. If they can’t, they’ll regard accepting help as an act of weakness and being dependent.” (Carer 7)
3. Interactions between patients and carers^Bo^	“We support each other on this journey. S/he (care-recipient) has persevered for so many years and that gives me confidence, a confidence that I can persevere like s/he does.” (Carer 3)
4. Support from other family members^Bo^	“Then they (patient and carer) have to find support from family members. Like the old couple, they may seek help from their children so that the carer can take a break occasionally.” (Professional 1)
5. Mutual support of carers^Bo^	“I finally identified a WhatsApp group of carers. We can share information and sometimes vent our emotions there.” (Carer 5)
6. Interpersonal network of carers^Bo^	“First, I will get to understand the case (carer) to see if any social support or network is available. Sometimes, if they need practical support, they can actually reach out to their friends or church members.” (Professional 20)
**Category 2: Social Care Services**	
7. Home-based support^Br^	“It takes 3 months before the domestic helper arrives. It’s better to have someone go to their homes and exchange dialysis fluid for them, rather than just sending them to elderly care homes.” (Professional 11)
8. Respite care^Br^	“The (day) hospital is good. It offers different activities and physiotherapy. They are indeed patients. We (carers) feel safe for them to be looked after by healthcare staff.” (Carer 20)
9. Venting space^Br^	“They are carers, so they have less time to keep their old friends. They have fewer and fewer friends over time. Then, they have fewer chances or channels to vent their feelings.” (Community Member 21)
10. Patient transport services^Br^	“We can recommend those accessible taxis or buses, but there are queues for transport services, and they are too expensive for them (patients and carers). They pay 100 dollars for a one-way trip.” (Professional 12)
11. Financial and material assistance^Br^	“Because of the household finances, they (patients and carers) need to work. If they can receive financial assistance, they can stay home and have more time to rest.” (Carer 9)
12. Timely transition to institutional care^Br^	“The biggest challenge is sustainability. They (carers) need to wait for 3 to 6 years for a place in an elderly home. They may put a lot of effort in caregiving at the beginning, but when time passes and they feel tired, some may want to withdraw from caregiving.” (Professional 16)
13. Need-oriented service design^Br^	“Although they (service providers) offer certain kinds of support or service, they don’t always meet the needs of carers. I think that understanding the needs of carers is of utmost importance.” (Community Member 8)
14. Carer-centered care approach^Br^	“The community resources for patients are much more than those for carers. Carers need to initiate supportive efforts by themselves.” (Professional 9)
15. Reaching out proactively^Br^	“If anyone wants to provide social capital or community support to these people (carers), they can’t say ‘I offer help, approach me.’ Instead, they need to reach out to these people, and tell them what support and service is available for them. This is the only way carers can find help.” (Community Member 8)
**Category 3: Healthcare Services**	
16. Optimizing treatments for patients^Br^	“If you can relieve the symptoms of the patient and improve their self-management and mental wellbeing, you can alleviate the burden of the carer.” (Professional 4)
17. Discharge support^Br^	“I heard a case that a physician discharged a patient without telling him what to do. Does that patient really know how he can manage his life at home?” (Carer 11)
18. Community-based healthcare^Br^	“In future, every public housing estate should have a center providing all kinds of (healthcare) services. They can offer basic care like the usual follow-ups. Of course, for more advanced care, you need to go to the hospital.” (Patient 11)
19. Healthcare professionals’ attitudes^Br^	“They show you that they are walking with you. They want to go through the journey with the patient and carer.” (Carer 2)
**Category 4: The Community**	
20. Mutual support of neighbors^Bo^	“If the carer wants to go out for grocery shopping, the neighbor may offer help to look after (the care-recipient) for a couple of hours. This is social capital, but I don’t think we can do this in Hong Kong. Even if we set up clinics inside the housing estate, the neighbors are not under your control.” (Community Member 18)
21. Trained volunteer workforce^Bo^	“The churches can help. There are many professionals attending churches. Carers can send their patients to churches for several hours. Without training, other people in the neighborhood do not dare to take up this responsibility.” (Community Member 5)
22. Flexible working arrangement^L^	“It’s (company) cold and unsympathetic. They knew my situation, but they didn’t show understanding and gave me more tasks to do. It’s still performance-driven. Even when I expressed my need to look after my hospitalized family member, my company still penalized me because I was late at work.” (Carer 11)
23. Inclusive public space^Br^	“We need to check the accessibility of exits when my mother needs to travel by train. If it’s far away, even if she wants to go out, she wouldn’t take this way.” (Carer 7)
24. Advocacy work^Br^	“If there is greater awareness in the community, people will be willing to help each other. Some normal people, who aren’t carers, may give a little help that solves a big problem for carers.” (Carer 5)
**Category 5: Information**	
25. Caregiving information^Br^	“In terms of practical support, they can teach you how to address the physical and psychological needs of patients with kidney failure. What do those signs and symptoms mean? I think there should be a full package, instead of just a lecture. A lecture is just a single session. I can forget everything 2 weeks after attending a talk.” (Carer 11)
26. Reliable and effective communication channels^Br^	“There is lots of information. You can find it on (social) media, on the internet, or on TV. Information is overwhelming nowadays. You can even find it on your cell phone.” (Patient 15)
27. Referral by social workers^Br^	“I know nothing about these resources. Are they really available? Medical social workers aren’t very caring. They manage financial assistance and conduct asset reviews. These tasks shouldn’t be the job of a social worker. What I need from them is information about community resources.” (Carer 11)
28. Healthcare professionals’ knowledge of social care^Br^	“They (patients and carers) believe in healthcare staff. It helps if physicians and nurses know more about the resources in community.” (Professional 15)
**Category 6: Policies**	
29. Increasing resources for carer services^L^	“There are too many time-limited projects. Sometimes, when they (carers) get to know the service, it has already ended. The situation here is that services are conducted on a project-by-project basis.” (Professional 16)
30. Coordinating supportive services^L^	“I think it’s (carer support) scattered. There are various types of carer support. We have many elderly centers, but how can they be coordinated? Is it the carer support that we want if something is offered to carers? Can carers find this support? Which organization they should approach?” (Professional 20)
31. Researching carer situation^L^	“We have started having some understanding about their (carers) situation, but that’s not enough. We don’t have figures and data to formulate policies that serve the public interest.” (Community Member 16)

*Bo* Bonding capital: *Br* Bridging capital: *L* Linking capital

### Carer attributes

Carer attributes are the qualities of carers that facilitate caregiving. These qualities can be regarded as a cognitive form of bonding capital that foster family caregiving. Some qualities are attitudes that facilitate the uptake of caregiving roles, such as a sense of responsibility and a readiness to accept support. The care recipient (i.e., the patient) is another source of support to the carer. A positive relationship between the patient and his/her carer is seen as a reward that supports caregiving.

Participants agreed that the social networks of carers could offer them different forms of continuous practical support. Of note, support from family members and other carers was deemed an important resource in caregiving. These individuals can assist in practical tasks, listen to the carers, and relay useful information.

### Social care services

Social care services, which are usually provided by NGOs in the community, remain a crucial part of carer support. Serving as the bridging capital between carers and social care professionals, participants highlighted the types and characteristics of these services that facilitate caregiving. Services that participants wanted included home-based support (e.g., meals-on-wheels, personal care, and household cleaning), respite care, psychological counselling, patient transport services, financial and material support, and institutional care that can be accessed within a short period. Regardless of the type of service, social care should be provided based on the actual needs of the carers, especially the unique needs of part-time or older carers who require additional support due to time constraints or disabilities.

While many services are designated to the patients, a model of care that centers on the carers is warranted. Because many carers lack information or networks informing them of the different services available, service providers who reach out proactively through door-to-door promotional campaigns, roadshows, or social media programs may improve service utilization.

### Healthcare services

Patients with AOF require continual medical care, and therefore, support from healthcare professionals is deemed crucial to patients and their carers. Some types and characteristics of healthcare services are deemed as the bridging capital between carers and healthcare professionals. Optimized treatments and discharge support are two elements of healthcare services that are important to carers. In addition, because many specialty services are currently situated in hospitals, patients need to be transported for follow-up appointments. Some participants expressed a need for community-based care, including some specialty services that can be shared by primary care providers.

In addition to the mode of delivery, some carers recalled their positive experiences of healthcare services that have been helpful to them. Healthcare professionals with caring and encouraging attitudes can motivate carers.

### The community

Interpersonal networks within a community (e.g., residents of a public housing estate) can serve as the bonding capital for carers. These networks often involve neighbors and volunteers who can provide immediate practical (e.g., grocery shopping or care replacement) and emotional support to carers. However, participants were concerned about their community members’ willingness and readiness to help, especially in Hong Kong. Relevant training prior to volunteering is warranted.

There are other characteristics of the community that can facilitate the social participation of carers, including inclusive public spaces and advocacy campaigns, which can be identified as different examples of bridging capital. Of note, many carers were still engaging in employment, and their employers’ understanding and provision of flexible working arrangements can foster a balance between family and career endeavors. These flexible working arrangements can become the linking capital that mobilizes organizational resources to foster caregiving.

### Information

The participants acknowledged that information circulated in mass and social media could empower carers. The expertise of professionals is shared through circulation of information, which can be deemed as an example of bridging capital. Information about health management, emergency handling, and social resources were in demand among the carers. While they could retrieve information from various channels, such as traditional media and interactive social media platforms (e.g., Facebook and WhatsApp), an integrated and reliable channel would significantly aid caregiving. Professionals, including physicians, nurses, and social workers, are regarded as reliable sources of information. While carers and patients believed in the expertise of these professionals in managing AOF, some participants stressed a need for them to have the most updated information on social resources.

### Policies

Government policies regarding carer support can be an important source of linking capital. Despite the availability of some supportive services, such as home-based support and respite care, policies guiding carer-centered services are lacking. While additional resources need to be set up to specifically promote the wellbeing of carers, better coordination between service providers may improve the coverage and continuity of these services. In addition, while the understanding of the needs of carers, especially in the local context, remains poor, some participants demanded additional research to generate evidence that could be used to develop comprehensive carer policies.

## Discussion

This study used a qualitative approach to outline the different sources of social capital available to carers of patients with AOF. Along with the previous information gathered on perceived social support [20, 21], the findings from this study add depth to our understanding of carer support. Based on a considerable number and range of participants from sectors across the community, six major sources were revealed. These sources contain bonding, bridging, and linking capital that extends the support network horizontally among carers’ families and neighbors and vertically across various social groups in the community. This comprehensive network can contribute to the sustainability of the social care systems involved in the provision of carer support.

The first category highlights the importance of enhancing the carer’s own capabilities in family caregiving. Factors such as the carers’ readiness, their relationship with the care recipient, as well as their interactions with other family members and carers can serve as intangible facilitators of caregiving. In one study [27], correlations were found between carer attachment (indicating the quality of the carer-care recipient relationship), self-efficacy (reflecting the carer’s perceived readiness), and carer burden. Despite the significance of these internal resources, current supportive services often overlook them and focus primarily on providing additional assistance. However, certain support group interventions that target self-efficacy have shown promising results in enhancing readiness and alleviating carer burden [28]. By fostering shared experiences and emotions, the formation of a caregiving network can not only provide valuable support but also enhance an individual’s capabilities for caregiving.

Our second and third categories suggest that while existing healthcare and social care services, such as home-based support and respite care, are crucial to caregiving, there are limitations that hinder their provision and utilization. This was echoed in a recent report [29], in which resources for carer support were found to be limited and loosely coordinated, and a more carer-centered focus was warranted. These limitations may be attributed to the current policies on carer support in Hong Kong [19]. Supportive services are currently provided under the themes of elderly care and rehabilitation (for the physically and mentally disabled), and the needs of the care recipients are often prioritized. The needs of carers are not formally recognized or proactively addressed, especially for those who take care of younger and less disabled patients with AOF. This situation is common worldwide, particularly in Asian countries such as India and Japan [29]. In fact, caregiving is a significant aspect of disease self-management [30]. Ongoing support from the healthcare team can lead to informed and motivated patients and carers who play an active role in the management of long-term conditions. Our findings reinforced the proposition that carers, together with the patients, should be placed at the center of care.

In the fourth category, we noticed that some emerging interpersonal networks are readily accessible to carers, including supportive relationships with neighbors and volunteers. However, as expressed by some stakeholders, some individuals may not feel prepared to support carers because of a lack of training and a sense of distance, which is a common phenomenon in a densely populated modern city like Hong Kong [31, 32]. As reported in previous studies [33], adequate skill training and emotional support are warranted to empower these individuals. These findings align with our fifth theme regarding information. Although carers often obtain information from mass media and professionals (e.g., social workers), there is a growing trend of connecting with others through online communities. This online connectivity can assist caregivers in accessing information and finding support. Evidence suggests that online communities may increase an individual’s sense of inclusion and belonging [34]. Therefore, researchers and service providers could explore how these communities can be incorporated into service models to enhance their reach and effectiveness.

**Fig. 1:**
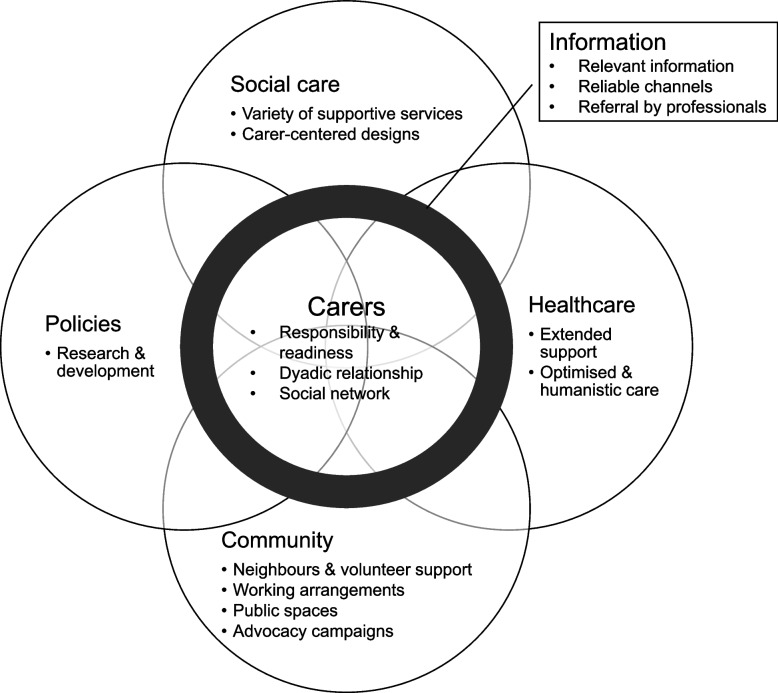
Layers of support surrounding the carers and care recipients, including family members and other carers, service providers, community, and society

In addition to supportive services and interpersonal networks, some characteristics of the community were perceived as social capital for family caregiving. For example, creating an inclusive environment that promotes both physical and social inclusion can enhance the participation of carers in their social roles. Implementing flexible working arrangements can assist carers in achieving a balance between their caregiving responsibilities and their careers. These characteristics are essential for creating a carer-friendly community [35, 36], yet underutilized and may be elicited by public education [37, 38] the support of relevant policies. In the last category regarding policies, stakeholders primarily focused on provision of carer-oriented services. According to the current policies, supportive services primarily focus on meeting the needs of care recipients, such as residential and respite care [19]. Consequently, stakeholders have expressed the need for more comprehensive and coordinated services specifically tailored for carers. In fact, findings from this qualitative study offer a comprehensive framework for enhancing support for carers of patients with AOF. Consistent with the Social Ecological Model [39], these different forms of social capital form layers of support through social interactions between individuals and social groups (Fig. 1). In the case of care for patients with AOF, carers can utilize internal resources to fuel their caregiving commitment; their interactions with social care and health care professionals to meet their practical needs; and the efforts of the community to mobilize individual or organizational resources to support caregiving. Policymakers identify needs and develop relevant carer support policies to address emerging needs. Together with information that connects these elements, they help constitute a positive environment for caregiving.

### Limitations

Several limitations warrant consideration. First, this study was conducted in a Chinese community, in which the caregiving context was influenced by the cultural value of filial piety [40]. The cultural context may indirectly affect the perception of caregiving-related responsibilities, although such influence has not been noted in current analysis. Second, given the study aims, individual perspectives were integrated into a comprehensive picture. Differences in the perspectives of stakeholders were not specifically examined. The findings from this study did not highlight the significance of any specific form or source of social capital. Third, because recruitment was disrupted by social distancing measures during the COVID-19 pandemic, relatively small numbers of patients with HF (*n* = 2) and few physicians (*n* = 1) were included in this study. However, based on data saturation and other measures of trustworthiness, the representativeness of the overall sample was guaranteed.

### Implications for policies

Based on the findings and discussions presented, several recommendations can be made to inform policy development and enhance support for carers of patients with AOF. Firstly, it is crucial to recognize and prioritize the needs of carers within existing healthcare and social care policies. The six categories provided a framework for formulating programs to support individual and social groups involved, from carers to service providers. A few specific findings warrant attention. There is a need to strengthen and expand existing supportive services. While adequate funding and improved coordination among various service providers are essential, innovative strategies, such as capacity building and online communities, should be incorporated into service models. In addition, public education and awareness campaigns should be launched to foster an inclusive and supportive community environment. Flexible working arrangements should also be encouraged to help carers strike a balance between their caregiving responsibilities and their careers.

### Implications for research

In view of the limitations, future research should prioritize cross-cultural comparisons to examine the influence of cultural values on carer support. It is important to explore diverse stakeholder perspectives, including carers, care recipients, healthcare professionals, and policymakers, to gain a comprehensive understanding of their needs and experiences. Utilizing participatory approaches, such as concept mapping [41], can facilitate active engagement of stakeholders in the research process. In addition, expanding sample sizes and conducting longitudinal studies can provide greater generalizability, allowing for a deeper exploration of the dynamic nature of social capital and the effectiveness of interventions.

## Conclusions

Many carers of patients with AOF experience a significant caregiving burden that leads to health problems and social isolation. Social capital, which is the internal strength of a community that facilitates different social roles, may support these carers. This qualitative study explored different sources of social capital from the perspectives of stakeholders. Six categories emerged, namely, carer attributes, social care services, healthcare services, the community, information, and policies. The findings suggest that a multi-layer support system exists in the community for carers to utilize. The community’s internal resources that support caregiving include personal attributes, interpersonal relationships, community resources, and societal contexts. While this system can serve as a framework for building a carer-friendly community, interventions may be required to strengthen some aspects of social capital.

### Supplementary Information

**Supplementary Material 1.**

## Data Availability

Data are available upon reasonable request to the corresponding author.
