# Supplementary material for: Social capital for carers of patients with advanced organ failure: a qualitative exploration of stakeholders’ perspectives
Source: BMC Public Health. 2024 Mar 2;24:670. doi: 10.1186/s12889-024-18213-6 (PMC10908001; doi:10.1186/s12889-024-18213-6)
Supplement: Supplementary file 1 — Supplementary Material 1. [file 12889_2024_18213_MOESM1_ESM.pdf]

INTERVIEW GUIDE

Introduction

Thank you for participating in this research study. Before the interview, I would like to introduce the concept of 'social capital.' Social capital refers to the standards, relationships, and norms that facilitate the interactions within a community. It includes attitudes of an individual, values of a group, and social systems that may improve the efficiency of society. Different forms of social capital exist, including visible features like participation in a social group and invisible features like a sense of trust. Social capital can be also categorised based on the direction of relationships. Some interactions link up individuals or groups with similar (e.g., peer support groups) or different (e.g., health care institutions) background, or even across the power difference (e.g., ways to communicate with the government). Today we would like to invite you to share what social capital may be used or strengthened to support carers of patients with advanced organ failure (i.e., chronic heart failure, chronic obstructive pulmonary disease, chronic renal failure).

Questions

1. Please generate some short sentences or phrases that describe the resources in the community, including, but not limited to, healthcare and social care services, that facilitate caregiving of patients.
2. In your experience/knowledge, what are the challenge faced by these carers? In terms of material needs? Psychosocial needs? Spiritual needs?
3. In the community (including family, neighbourhood, social and medical care services, and the society as a whole), what can support these carers to overcome the challenges you mentioned?
4. Do you think this support is adequate? What is the room for improvement?

Conclusion

Thanks again for your participation.
